# Distribution of hospital care among pediatric and young adult Hodgkin lymphoma survivors—A population‐based cohort study from Sweden and Denmark

**DOI:** 10.1002/cam4.2363

**Published:** 2019-07-02

**Authors:** Ingrid Glimelius, Annika Englund, Klaus Rostgaard, Karin E. Smedby, Sandra Eloranta, Peter de Nully Brown, Christoffer Johansen, Peter Kamper, Gustaf Ljungman, Lisa Lyngsie Hjalgrim, Henrik Hjalgrim

**Affiliations:** ^1^ Department of Immunology Genetics and Pathology Uppsala University Uppsala Sweden; ^2^ Division of Clinical Epidemiology, Department of Medicine Karolinska Institutet Stockholm Sweden; ^3^ Department of Women's and Children's Health Uppsala University Uppsala Sweden; ^4^ Department of Epidemiology Research Statens Serum Institut Copenhagen Denmark; ^5^ Department of Haematology Rigshospitalet Copenhagen Denmark; ^6^ Danish Cancer Society Research Center University of Copenhagen Copenhagen Denmark; ^7^ Department of Haematology Aarhus University Hospital Aarhus Denmark; ^8^ Department of Paediatrics and Adolescent Medicine Rigshospitalet Copenhagen Denmark

**Keywords:** Hodgkin lymphoma, hospitalizations, late adverse effects, relapse, survivorship

## Abstract

The burden of late effects among Hodgkin lymphoma (HL) survivors treated according to contemporary protocols remains poorly characterized. We used nation‐wide registers to assess number of inpatient bed‐days and specialist outpatient visits among 1048 HL‐patients (<25 years, diagnosed 1990‐2010) and 5175 country‐, sex‐, and age‐matched comparators. We followed them for up to 24 years, with time‐dependent assessment of relapse status. International Classification of Diseases (ICD‐10) chapter‐specific hazard ratios (HRs) were assessed in Cox regression analyses, and nonparametric statistics described patterns of health‐care‐use. Relative to comparators, *relapse‐free survivors* were at increased risk of infections, diseases of the blood, endocrine, circulatory and respiratory systems, and unspecific symptoms, HRs ranging from 1.86 to 3.05. Relative to comparators, *relapsed survivors* had at statistically significantly increased risk of diseases reflecting practically all investigated disease‐chapters, HRs ranging from 1.60 to 18.7. Among *relapse‐free survivors,* 10% of the patients accounted for 80% of all hospital bed days, and 55% were never hospitalized during follow‐up. Among *relapsed‐survivors*, 10% of the patients accounted for 50% of the bed days, and only 24% were never hospitalized during follow‐up. In contrast, 10% of the comparators accounted for 90% of hospital bed days and 75% were never hospitalized. These findings challenge the impression of a uniformly distributed long‐term morbidity among all HL survivors and emphasize the need for early identification and attention to patients particularly susceptible to late effects, such as relapsed survivors.

## 1 INTRODUCTION

Modern therapy for Hodgkin lymphoma (HL) offers cure rates exceeding 90%.^1^, ^2^, ^3^ A general impression is that a high price for HL cure entails of a high risk of adverse treatment effects.^3^, ^4^, ^5^, ^6^ Consequently, endeavors are continuously ongoing to define treatment regimens that have fewer late effects while maintaining the high cure rates.^7^

Numerous investigations have addressed risk factors for late morbidity among survivors of cancer at young ages.^8^, ^9^, ^10^, ^11^, ^12^, ^13^ While the spectrum of late effects of treatment is likely to vary among HL survivors treated before and after the early, or mid 1990s owing to changes in therapy,^14^, ^15^ it is clear from literature that morbidity is elevated among HL survivors compared with the general population, and that the risk depends on intensity and type of treatment. However, although relevant both to patients and for health care planners, little is known about how this disease burden is distributed among survivors, particularly not between relapsing and relapse‐free patients.

Efforts to identify late effects from treatments may benefit from observational studies^16^ when population‐based cohorts with long‐term follow‐up and complete coverage of outcome data exist.

In addition, treatment comparisons are possible if administrative circumstances dictate protocol choice, as is often the case for HL among adolescents and young adults (AYA). For instance, both in Sweden and Denmark patients at opposite ends of the AYA age spectrum are treated according to pediatric and adult protocols, respectively, differing with regard to both drugs used and radiation criteria. At the same time, radiotherapy has historically been more common in young Swedish HL patients compared to Danish HL patients, adding another dimension to treatment variation.^17^

To advance the understanding of HL survivor morbidity, we assessed use of out‐ and inpatient care in a population‐based contemporarily treated cohort of children, adolescents, and young adults diagnosed with HL in Denmark and Sweden with detailed information on treatment and relapse and contrasted with a matched sample of general population comparators.

## 2 MATERIALS AND METHODS

### 2.1 Study population, comparators, and setting

Our study cohort has been described previously.^2^, ^17^, ^18^ Briefly, through hospital file review and population‐based hospital‐, cancer‐, and lymphoma registers we identified all individuals diagnosed with HL before the age of 25 years in the period 1992‐2009 in Sweden and 1990‐2010 in Denmark. Available data included information on nationality, gender, date of birth and diagnosis, Ann Arbor disease stage at diagnosis, primary treatment and outcome, and when present and relevant, relapse treatment, and outcome. Treatment information for Danish children was from medical records, for Swedish children from the Swedish Childhood Cancer register and for adults in both Denmark and Sweden treatment information came from the Nationwide lymphoma registers. In addition, missing information was in selected cases identified through medical record review and added to the lymphoma registers and Swedish Childhood Cancer register prior to linkage to the cause of death and national hospital registers. For each patient we identified up to five comparators in the Swedish and Danish population‐registers, respectively, who were matched to the index patient on country, sex and age at diagnosis, and alive and free from HL at diagnosis of the index patient.^19^, ^20^ The matched comparators were followed from the corresponding diagnosis date of the index patient.

### 2.2 Outcomes

Using the personal identification number unique to all individuals in Sweden and Denmark, we linked the cohorts of HL patients and comparators to national population—and cause of death registers^19^, ^20^ to ascertain vital status, to national hospital registers^21^, ^22^ to ascertain information on hospital care following HL treatment and corresponding time windows for the comparators, and to the national cancer registers^23^, ^24^ to ascertain secondary malignancies among the HL patients.

The outcome data was retrieved for the calendar years 1994‐2014 (Denmark) and 1997‐2012 (Sweden), defining the (country‐specific) study periods when inpatient and specialist outpatient diagnosis registration were nation‐wide and according to the International Classification of Diseases revision 10 (ICD 10) in both countries. In Sweden information on diagnoses relating to outpatient visits was available only from 2001, and accordingly the Swedish contribution to the outpatient visit analyses was restricted to the period 2001‐2012. We grouped diseases in inpatient and outpatient registrations according to ICD chapters,^25^ excluding diagnoses in chapters XV, XVI, XVII, XX, XXI, XXII: that is, diagnoses related to pregnancy, malformations, the perinatal period, and external causes of morbidity, since we did not consider those as treatment complications^26^ (Table S1). The disease group: “Symptoms” includes symptoms, signs or abnormal clinical and laboratory findings not elsewhere classified. Furthermore, we disregarded all outpatient visits with HL/HL relapse or non‐HL (C81‐C85) as main diagnosis (assumed to represent clinical check‐up visits). Finally, we aggregated chapters VI, VII, and VIII under the heading central nervous system (CNS) morbidity due to small numbers.

### 2.3 Follow‐up

We followed patients and comparators from time of primary HL diagnosis or start of study period, whichever occurred last, until the end of the study period, death or the relevant outcome in incidence analyses, whichever occurred first.

We stratified patients according to baseline characteristics and first‐line treatment modalities. We assumed primary HL and HL relapse treatment took place in the 1‐year period following the diagnosis or relapse to distinguish hospitalizations related to HL treatment. Thus, we stratified follow‐up time according to time since primary diagnosis (0, 1‐3, 4‐6, 7‐9, 10‐12, 13+ years), and according to time since first relapse, that is, 0, 1+ years since relapse. Using combinations of these time intervals (states) patients were time‐dependently grouped into four strata: relapse‐free patients under treatment, relapse‐free patients post treatment, denoted *relapse‐free survivors*, relapsed patients under relapse treatment and relapsed patients post‐relapse treatment, denoted *relapsed survivors*. *Comparators* and their follow‐up time were assigned to the same stratum as their index person to allow comparison with the background population. Thus, any patient or their matched comparators would contribute follow‐up time and outcomes to at least one of these four strata, and at most all four strata during follow‐up, but only to one stratum at a time.

**Figure 1:**
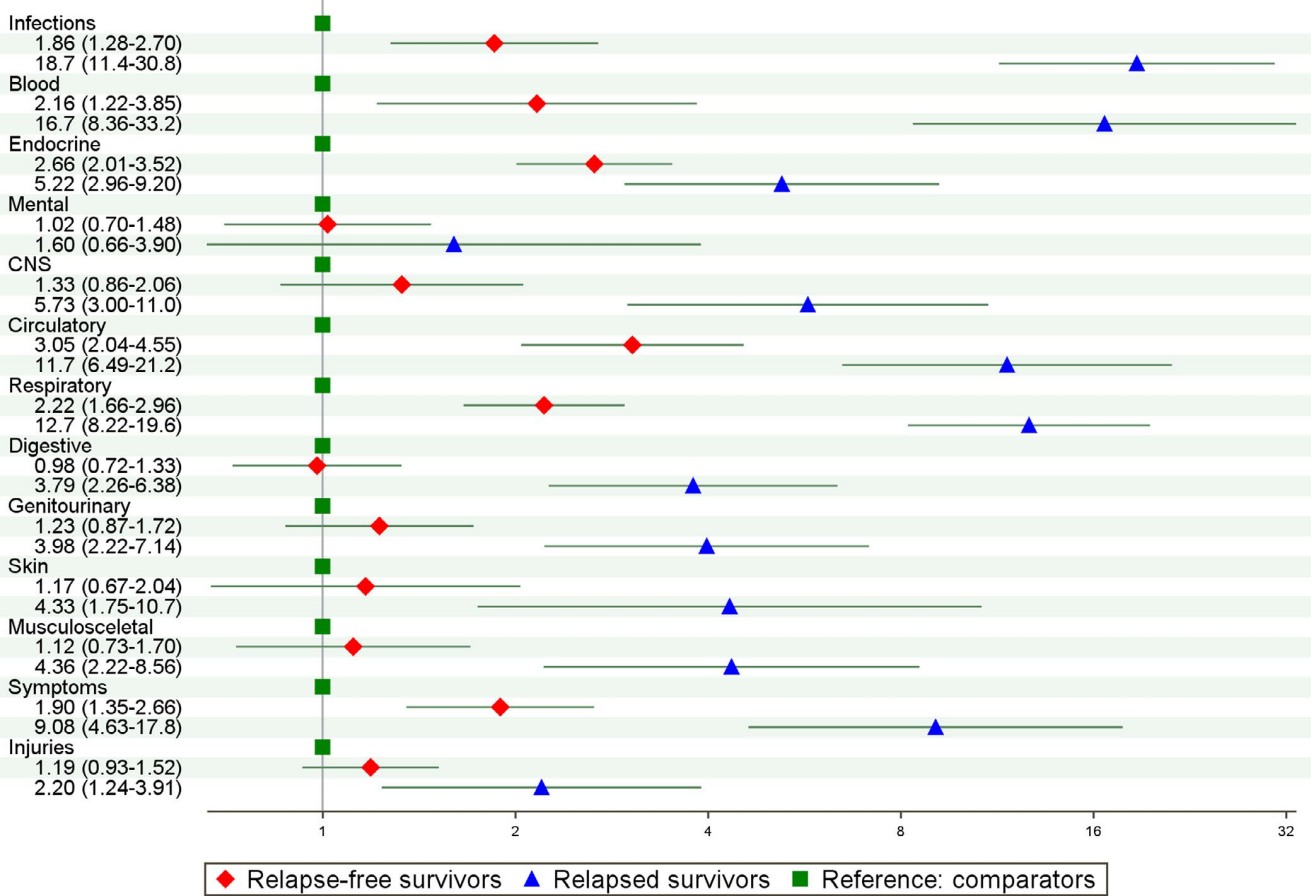
Hazard ratios with 95% confidence intervals (CI) for inpatient hospitalizations due to specific disease‐chapters for Hodgkin lymphoma patients and comparators, stratified by relapse status. Hazard ratios for incidence of inpatient hospitalization by International Classification of Diseases (ICD)‐chapters for Hodgkin lymphoma patients (<25 y) diagnosed 1990‐2009 in Sweden and Denmark and matched comparators. Relapse‐free survivors are indicated by red diamonds, relapsed survivors by blue triangles and comparators, the reference group by green squares. Lines indicate 95% CI. Specific ICD‐codes are indicated in Table S1. Abbreviations: Blood, Blood disorders; CNS, Central Nervous system disorders; Symptoms, Unspecified symptoms

**Figure 2:**
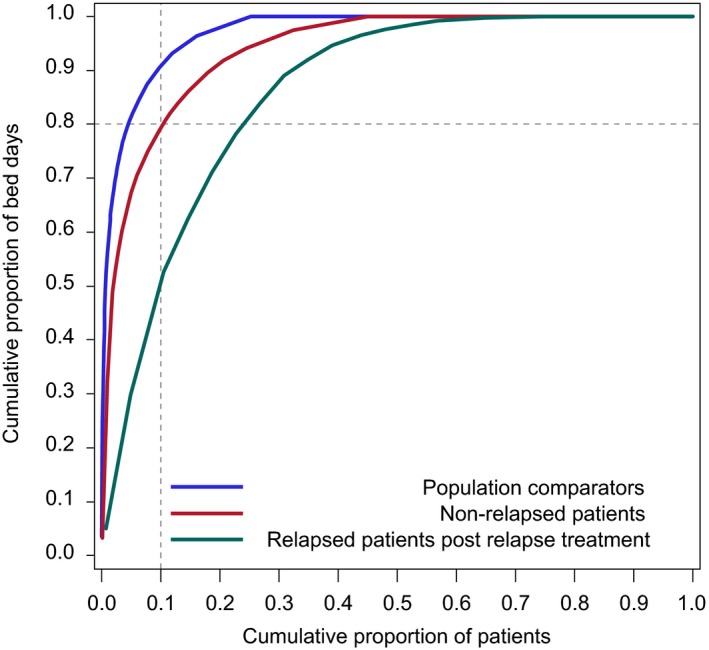
Inpatient hospitalizations for Hodgkin lymphoma (HL) patients and comparators, stratified by relapse status. Inverted Lorenz curve showing inverted cumulative percentage frequency distributions of bed days spent in hospital by HL relapse‐free survivors, relapsed survivors and population‐comparators (see text for definitions). The X axis shows decreasing deciles of bed days spent in hospital during the entire follow‐up by members of the respective cohorts and the Y axis the cumulative proportion of all bed days in hospital during the entire follow‐up for the entire cohort that are accounted for by patients at the relevant decile. Hospital contacts due to pregnancy, childbirth, conditions in the perinatal period and congenital malformations were ignored. The reference line illustrates that the 10% of the individuals who had spent most days in hospital accounted for approximately 90%, 80%, and 50% of the total number of bed days accumulated by the comparators, relapse‐free, and relapsed survivors, respectively

**Figure 3:**
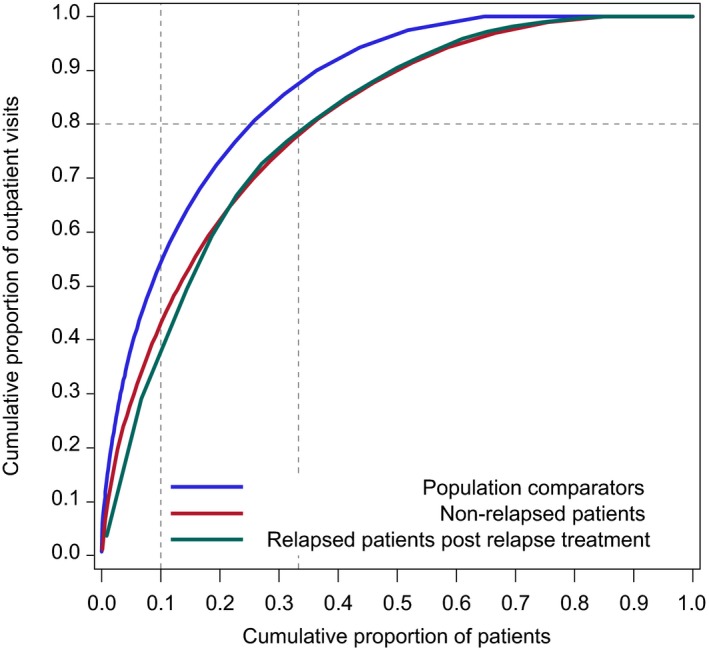
Specialist outpatient visits for Hodgkin lymphoma (HL) patients and comparators by relapse status. Inverted Lorenz curves showing inverted cumulative percentage frequency distributions of number of outpatient visits paid by HL relapse‐free survivors, relapse survivors, and population‐comparators (see text for definitions). The X axis shows decreasing deciles of outpatient visits by members of the respective cohorts and the Y axis the cumulative proportion of all outpatient visits for the entire cohort that are accounted for by patients at the relevant decile. Outpatient contacts only due to pregnancy, childbirth, conditions in the perinatal period and congenital malformations were ignored. The reference line illustrates that the third of the most frequently admitted individuals accounted for 90%, 80%, and 80% of all outpatient visits accumulated by the comparators, relapse‐free, and relapsed survivors, respectively

**Figure 4:**
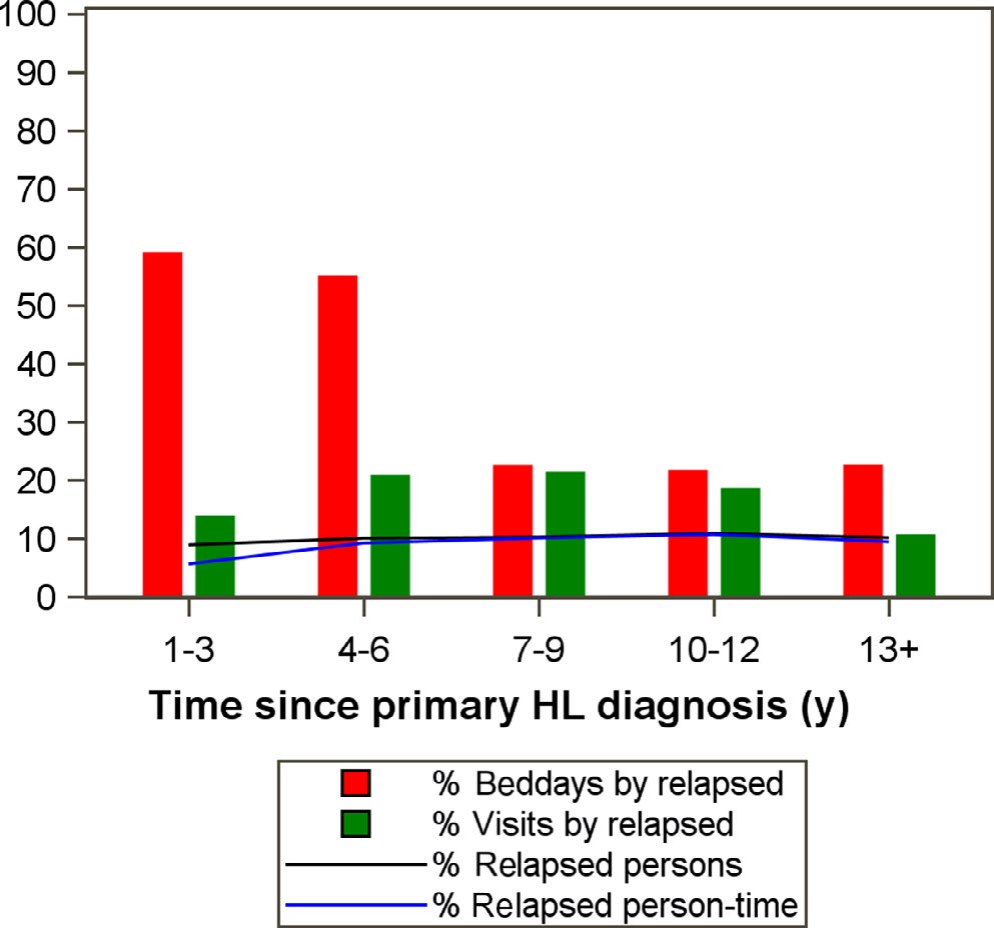
Number and proportion of patients contributing to used bed days and outpatient visits. Percentage of patients contributing person time and proportions of bed days (red bars) and specialist outpatient visits (green bars) stratified by follow‐up time for relapse survivors. Relapse‐free survivors contributed to the rest of the bed days and outpatient visits adding up to a 100% (see text for definitions of relapsed and relapse‐free) patients. The 10% of the patients with a relapse contributed to about a half of the number of bed days the patients used during years 1‐6 and about 20% from year 7. They also contributed to about 20% of the outpatient visits except during the first 3 y and after 13 y of follow‐up

We prepared outcome data for two distinct types of analyses assessing (a) incident outcomes, that is, first occurrence of diagnoses in broad groups (Figures 1 and [Link], [Link], [Link], [Link]), and (b) descriptive characteristics of hospital use based on total number of inpatient admissions and bed days and outpatient visits per time‐period of follow‐up (Tables 2 and S2; Figures 2, 3, 4).

### 2.4 Statistical analyses

Comparisons of incidence rates of ICD chapter‐specific diseases among different patient groups (including population controls) in terms of hazard ratios (HRs) and 95% confidence intervals (CI) were performed as a series of independent unadjusted Poisson regression analyses over follow‐up time intervals defined by time since primary HL diagnosis. The incidence of non‐HL malignancies after diagnosis/pseudo‐diagnosis in patients and controls was analyzed using Poisson regression, presenting hazard ratios with likelihood‐ratio based confidence intervals. Follow‐up was from diagnosis/pseudo‐diagnosis or 1 January 1994 (Denmark) or 1 January 1997 (Sweden), whichever occurred later until the occurrence of malignancy studied, death, or end of study, whichever occurred first.

Comparisons regarding number and length of inpatients admissions, bed days and number of outpatient visits and derivatives thereof were descriptive and nonparametric.

We chose to illustrate the distribution of hospital bed days and outpatient visits among the HL patients and comparators by means of Lorenz curves,^27^ traditionally used to display inequality in the distribution of income, wealth or other resources in a population. For reasons of presentation we have inverted one of the axes in this construction, thereby in Figures 3 and 4 producing “inverted Lorenz curves” regarding the use of bed days and outpatient visits. The information content is the same as for the original Lorenz curves.

All analyses were performed in SAS version 9.4.

## 3 RESULTS

Overall, we followed 1048 HL patients and 5175 country‐, sex‐, and aged‐matched comparators (Table 1). Overall there were equally many male and female patients. There were more males than females in the youngest age groups (patients treated in pediatric departments), and, conversely, more females than males in the older age groups (patients treated in adult departments). Slightly less than half (47%) of the patients presented with limited stage disease (I‐IIA) (Table 1). Again, there was some variation by age with advanced stage (IIB‐IV) being most common in the young adult group.

**Table 1:** Characteristics of patients and matched comparators, showing person years and numbers contributing to a given follow‐up stratum

Baseline characteristics	All patients		Pediatric Dept^a^ 0‐15 Den 0‐18 Swe		Adult Dept^a^ 15‐25 Den, 18‐25 Swe		Comparators^b^	
	N (%)	PY	N (%)	PY	N (%)	PY	N (%)	PY
Overall	1048	11591	318	3469	730	8122	5175	56492
Mean follow‐up overall (Years)		(11.1)		(10.9)		(11.1)		(10.9)
Gender								
Females	524 (50)	5622	147 (46)	1613	377 (52)	4009	2585 (50)	27859
Males	524 (50)	5969	171 (54)	1856	353 (48)	4113	2590 (50)	28633
Country								
Denmark	450 (43)	5429	90 (28)	1012	360 (49)	4416	2250 (43)	26361
Sweden	598 (57)	6163	228 (72)	2457	370 (51)	3706	2925 (57)	30131
Stage								
I‐IIA	492 (47)	5650	156 (50)	1693	336 (46)	3957	—	—
IIB‐IV	547 (52)	5822	159 (50)	1737	388 (54)	4084	—	—
Radiotherapy (RT)								
No RT	335 (34)	3434	110 (36)	1092	225 (33)	2341	—	—
Given RT	646 (66)	7636	193 (64)	2250	453 (67)	5386	—	—
Chemotherapy								
2‐4 courses	465 (48)	5001	222 (71)	2298	243 (37)	2703	—	—
6‐8 courses	499 (52)	5705	90 (29)	1096	409 (63)	4608	—	—

Time dependent characteristics^c^	All patients		Pediatric Dept^a^ 0‐15 Den 0‐18 Swe		Adult Dept^a^ 15‐25 Den, 18‐25 Swe		Comparators^b^	
	N (%)	PY	N (%)	PY	N (%)	PY	N (%)	PY
Relapsed^d^								
No	1039 (88)^d^	10539	317 (89)	3197	722 (88)	7342	—	—
Yes	140 (12)	1052	40 (11)	273	100 (12)	780	—	—
Time since diagnosis^b^ (years)								
0	910 (20)	885	269 (19)	258	641 (20)	627	4490 (20)	4369
1‐3	1012 (22)	2772	302 (21)	817	710 (22)	1955	5000 (22)	13741
4‐6	965 (21)	2608	287 (20)	769	678 (21)	1840	4773 (21)	12874
7‐9	777 (17)	2044	231 (16)	621	546 (17)	1423	3832 (17)	9954
10‐12	587 (13)	1473	185 (13)	473	402 (12)	1000	2817 (12)	7026
13+	390 (8)	1808	132 (9)	532	258 (8)	1277	1845 (8)	8529

Abbreviations: Den, Denmark; N, Number; PY, person years of follow up; Swe, Sweden.

a The age limit is set to 15 years in Denmark and 18 years in Sweden due to treatment traditions in the respective countries. These country‐specific administrative boundaries between pediatric and adult departments are rarely violated in clinical practice.

b For comparators diagnosis should be interpreted as index date.

c The number of relapsed and non‐relapsed patients adds to > 1048 and change over time since a person start as non‐relapsed and move to the relapsed group at the date of relapse, the numbers indicate the number of individuals contributing to each given cell. The corresponding comparators move group as their corresponding case move group.

d Nine patients relapsed before start of follow‐up explaining the reduction from 1048 to 1039 in the time dependent characteristics.

Overall, 140 patients (12%) experienced relapse following primary treatment, including nine patients who did not respond during primary treatment or relapsed within 3 months and were considered primarily progressive. For relapsing patients, the median time from diagnosis to relapse was 1.1 years (range: 0.1‐16.5) and the median follow‐up time from relapse was 6.7 years (range: 0.1‐20.0).

### 3.1 Frequencies of hospitalizations

We characterized patterns of hospital care by tabulating frequencies of hospital admissions and their durations for comparators, relapse‐free, and relapsed survivors in periods more than 1 year after primary diagnosis or relapse‐diagnosis (Table 2). Among the comparators 25% had one or more hospitalizations during follow‐up while 75% were never hospitalized. Among relapse‐free 45% had one or more hospitalizations and 55% were never hospitalized. Among relapsed 76% had one or more hospitalization more than one year after primary‐ or relapse‐diagnosis and only 24% were never hospitalized. In addition, except for individuals experiencing more than 10 hospitalizations during follow‐up, hospitalizations tended to be longer for relapsed survivors than for comparators and relapse‐free survivors (Table 2).

**Table 2:** Distributions of hospitalizations for patients stratified by relapse status and comparators

Hospitalization frequency	N of patients with different number of hospitalizations (%)			Mean number of bed days (mean number of bed days per hospitalization)		
	Comparators	Relapse‐free^a^	Relapsed	Comparators	Relapse‐free^a^	Relapsed
0 hospitalization	3832 (75)	533 (55)	30 (24)	0	0	0
1 hospitalization	706 (14)	199 (20)	22 (18)	2 (2.0)	2 (2.2)	3 (3.0)
2‐4 hospitalizations	445 (9)	165 (17)	21 (17)	7 (2.7)	7 (2.7)	11 (3.8)
5‐9 hospitalizations	93 (2)	48 (5)	17 (14)	26 (4.2)	24 (3.7)	36 (5.3)
10+ hospitalizations	49 (1)	26 (3)	33 (27)	124 (6.0)	77 (4.9)	92 (4.1)
**1+ (one or more hosp.** b **)**	**1293 (25)**	**438 (45)**	**93 (76)**	**10 (3.8)**	**11 (3.5)**	**42 (4.2)**

Number (left three columns) and length (right three columns) of inpatient admissions more than one year after primary or relapse diagnosis.

Among the comparators 25% had one or more hospitalization during follow‐up while 75% were never hospitalized. Among relapse‐free 45% had one or more hospitalization and 55% were never hospitalized. Among relapsed 76% had one or more hospitalization more than 1 y after primary‐ or relapse‐diagnosis and 24% were never hospitalized.

Abbreviations: N, Number.

a Please note that patients start in the not relapsed group and move to the relapsed group at the date of relapse.

b 1+ (a combined group of patients having one or more hospital inpatient admission).

### 3.2 Second cancers among patients

Among the HL patients, 35 experienced secondary malignancies, corresponding to a hazard ratio compared to controls of 3.00 with 95% CI of 1.95‐4.54. The secondary malignancies included six cases of cervix cancer (HR = 1.04;95% CI:0.39‐2.35), six cases of breast cancer (14.7;3.38‐100), three cases of myelodysplastic syndrome or acute myeloid leukemia (14.6;1.86‐296) and nine cases of skin cancer (4.89;1.91‐12.5). Six HL patients experienced a secondary malignancy after a relapse (9.30;2.66‐36.4).

### 3.3 Risk of disease‐specific hospitalizations

We compared incident discharge diagnoses grouped by ICD‐10 chapters between comparators, relapse‐free, and relapsed survivors (Figure 1). These analyses showed that relative to comparators, relapse‐free survivors were at increased risk of inpatient hospitalizations for infections, for diseases of the blood, endocrine, circulatory, and respiratory system disorders and for unspecified symptoms. Meanwhile, relative to comparators relapsed survivors had increased risk of being hospitalized for conditions across the entire spectrum of diseases with the exception of mental disorders (Figure 1).

Comparisons between patients with different disease stages, reflecting also the burden of therapy (Figure S1) or types of treatment: pediatric vs adult department (Figure S2), radiotherapy vs no radiotherapy (Figure S3) and 1‐4 cycles of chemotherapy vs 5‐8 cycles of chemotherapy (Figure S4) produced mostly inconspicuous/small differences.

### 3.4 Distribution of hospital care among patients and comparators

We illustrated use of hospital care in the period more than 1 year after diagnosis or relapse diagnosis by showing distributions of bed days and outpatient visits, respectively, for each of the studied cohorts (Figures 2 and 3).

Hospital care was unevenly distributed among both comparators, relapse‐free, and relapsed survivors with a small proportion of individuals accounting for most of the respective cohorts' total (cumulative) number of bed days and outpatient visits, respectively (Figures 2 and 3). However, the degree of unevenness differed between patient groups and comparators. For example, the 10% of the individuals (supportive line in Figure 2) who had spent the most days in hospital accounted for approximately 90%, 80%, and 50% of the total number of bed days accumulated by the comparators, relapse‐free, and relapsed survivors, respectively. Similarly, the one‐third (supportive line in Figure 3) of the most frequently admitted individuals accounted for 90%, 80%, and 80% of all outpatient visits accumulated by the comparators, relapse‐free, and relapsed survivors, respectively.

We next tabulated all bed days and outpatient visits for comparators, relapse‐free, and relapsed survivors in different time intervals since primary diagnosis. As shown in Figure 4 and Table S2, despite being few (10%), the relapsed survivors accounted for a disproportionally large burden of health care use as reflected in number of days in hospital (bed‐days) as compared to all relapse‐free (90%) survivors, especially from 1 up to the first 6 years after primary diagnosis. Similarly, relapsed survivors also accounted for a disproportionate number of out‐patient visits in all follow‐up periods except possibly 13+ years after primary diagnosis (Figure 4).

## 4 DISCUSSION

We followed a cohort of more than 1000 HL patients diagnosed before the age of 25 years to characterize their morbidity more than 1 year after primary diagnosis or relapse‐diagnosis. In agreement with similar studies, we showed that compared with the general population, HL survivors are at increased risk of being hospitalized for a wide array of diseases. However, our investigation expands the understanding of HL survivor morbidity by demonstrating that especially patients surviving relapsed disease were at greater risk and had more and longer hospitalization than relapse‐free survivors during follow‐up. In addition, among relapse‐free survivors, we demonstrate that disease stage at diagnosis was of limited significance for later morbidity. A small proportion of relapse‐free survivors accounted for many hospital contacts and likely need care, and close follow‐up, but indeed more than half of the relapse‐free survivors were never hospitalized during follow‐up.

We are unaware of other investigations that have provided similarly detailed insight into the distribution of hospital care among young HL survivors. Hospitalization rates have previously been used as a measure of the burden of late effects of treatment among young cancer survivors,^8^, ^9^, ^10^, ^28^, ^29^, ^30^, ^31^, ^32^ although only few studies have focused specifically on HL patients.^10^, ^12^, ^26^, ^33^, ^34^ Our finding that the excess hospital use among survivors is mainly driven by the relapsing individuals is in line with a few other previous investigations.^26^, ^35^, ^36^ Results similar to the present study were recently reported in a Danish population‐based study of 1768 5‐year survivors of HL, diagnosed at ages 15‐39 years in the period 1943‐2004.^37^ That study overlapped with the present investigation for the subset of 15‐24‐year‐old Danish patients diagnosed between 1990 and 2004, who survived their disease by 5 years or more, but included neither children, nor outpatient data or clinical information, such as disease stage, and further, only approximated relapse‐status. The calendar years in that study also covered treatments that are today outdated.

Our analyses highlight what may only be implicitly understood from previous studies. Specifically, if number of bed days is interpreted as a measure of morbidity, the clinical implication learned is that a small subset of the survivors appears to be particularly susceptible to late effects of treatment.^26^ Although the general burden of disease was higher in relapse‐free survivors in comparison with the general population, more than half of this population was never hospitalized during the follow‐up period. Therefore, for most HL survivors, the risk of severe morbidity requiring inpatient care may be lower than generally assumed and this message is important to communicate to HL patients, families, and caregivers. Our observation of most of the morbidity pertaining to a small minority of the HL patients would in all likelihood be supported a fortiori if the patients with the worst prognosis could somehow be salvaged from death and thereby contribute more to our tables and figures.

The present investigation was also inspired by differences in HL treatment dictated by administrative and geographical circumstances, with Swedish children receiving more radiotherapy than Danish children.^17^ Interestingly, however, we saw little evidence that these differences were reflected in differences in admissions to inpatient and specialist outpatient care during our follow‐up span. Thus, the different treatment recommendations in the countries did not influence the burden of late effects. However, despite the rather long follow‐up of our investigation, interpretational caution is still warranted due to the long lag‐time between given radiotherapy and severe late adverse effects, such as secondary malignancies and cardiovascular diseases.^5^, ^6^, ^12^, ^38^

Avoiding a relapse is important not only for the individual patient and for immediate survival, but also for future fertility in survivors^39^ and from a public health‐care perspective. Regarding follow‐up recommendations for HL patients, a broad spectrum of diseases seen for relapsing patients show the importance of awareness of many side effects, a broad follow‐up program and communication between hematologist/oncologists and other health care providers. An increase in risk was seen for infections, blood, endocrine, circulatory, and respiratory disorders also in relapse‐free, confirming these well‐known treatment side effects and supporting current follow‐up recommendations for them.

So far, no randomized trials have investigated differences in outcome and late effects between adult and pediatric treatment protocols^40^ and, since HL particularly affects the AYA age spectrum, interdisciplinary collaborations might further optimize their treatment.^41^ There are a few studies including adolescents in adult trials,^42^, ^43^ with satisfying treatment results, one describing no difference in secondary malignancies (16‐21 vs 22‐45 years), another noting them to be more frequent in young adults (21‐45 vs 15‐20 years). One study has described better event‐free survival and overall survival in patients <18 years treated according to pediatric protocols,^44^ with no data on late adverse effects. The lack of any significant differences in frequency of late adverse effects in the first decade after primary treatment among patients treated in pediatric and adult departments and also relatively few secondary malignancies indicates that the different strategies result in the same long‐term outcome. However, since some late‐effects, in particular secondary malignancies, have an incubation period of 20‐30 years, we cannot rule out that differences will eventually emerge.

Our investigation has several strengths but also limitations. A major strength is its population‐based approach with available detailed clinical information on patients treated according to modern protocols. We also relied on high quality registers for outcome ascertainment as almost all in‐ and outpatient specialist care in Sweden and Denmark are publicly funded and therefore subject to mandatory documentation. Weaknesses include that we were not able to reliably identify second or third relapses, which probably account for some of the excess hospital care required by the relapsing patients. However, the differences observed between relapse‐free and relapsed patients may also be underestimated due to a higher mortality among the relapsed patients. Finally, we limited follow‐up to periods when registers in both countries used ICD10 classifications. Because a proportion of patients were diagnosed before the introduction of the classification, our analyses are encumbered by an element of left‐truncation.

## 5 CONCLUSIONS

The majority of survivors after young HL had few or no inpatient bed‐days after the primary treatment, whereas a small number of individuals were heavily burdened by late morbidity. Patients surviving disease relapse accounted for a disproportionately large share of bed‐days accrued by the entire HL patient cohort. Relapse‐free patients with different initial stages, different treatment concepts and treatment in a pediatric or adult department had on the other hand very similar future late morbidities. Relapse and the consequences from relapse treatment seem most important to avoid also from a future health care use perspective and these patients need extra attention during follow‐up.
