# Supplementary figures and images for: Distribution of hospital care among pediatric and young adult Hodgkin lymphoma survivors—A population‐based cohort study from Sweden and Denmark
Source: Cancer Med. 2019 Jul 2;8(10):4918–27. doi: 10.1002/cam4.2363 (PMC6712477; doi:10.1002/cam4.2363)

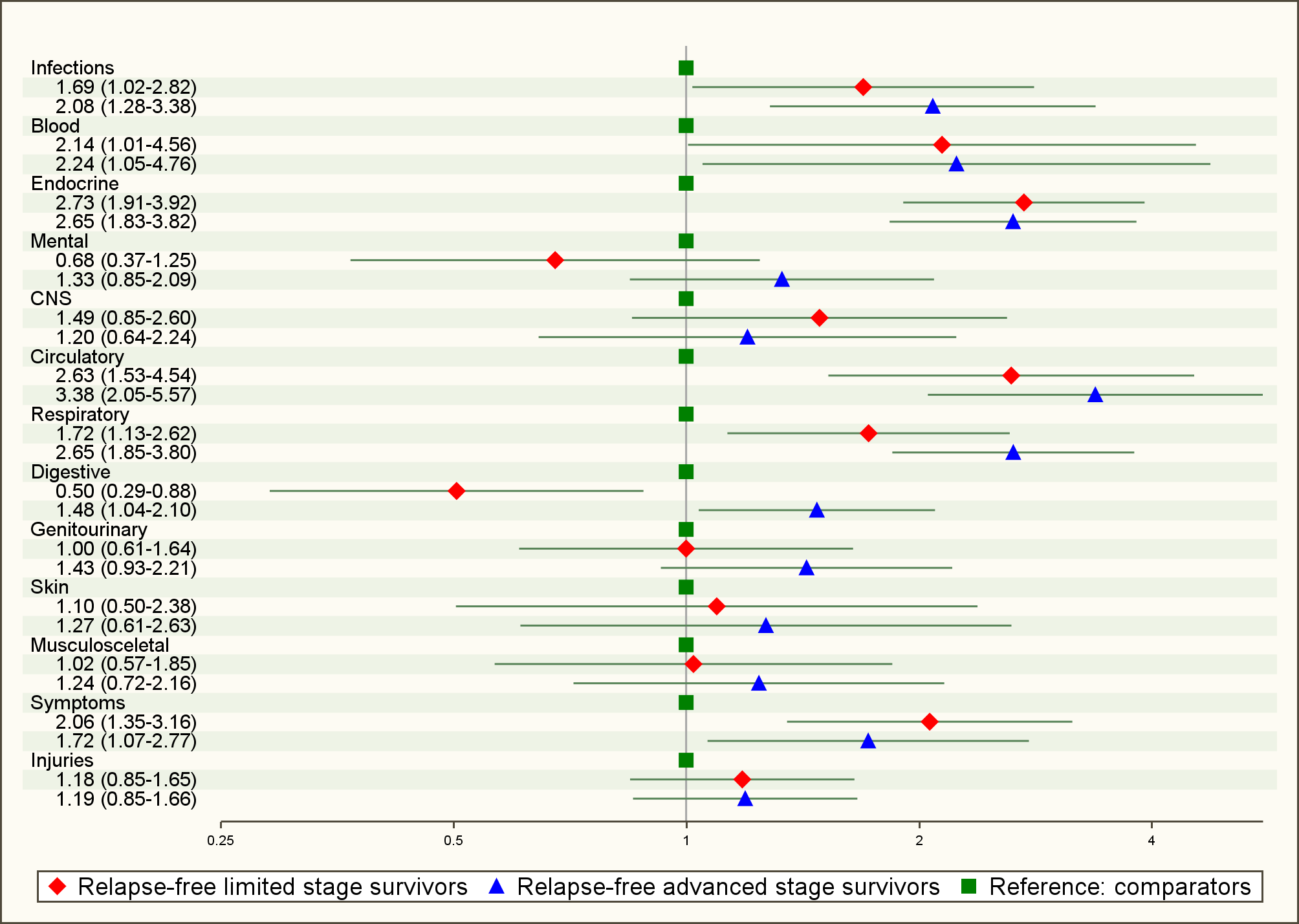

Supplement: Supplementary file 1 [file CAM4-8-4918-s001.tiff]

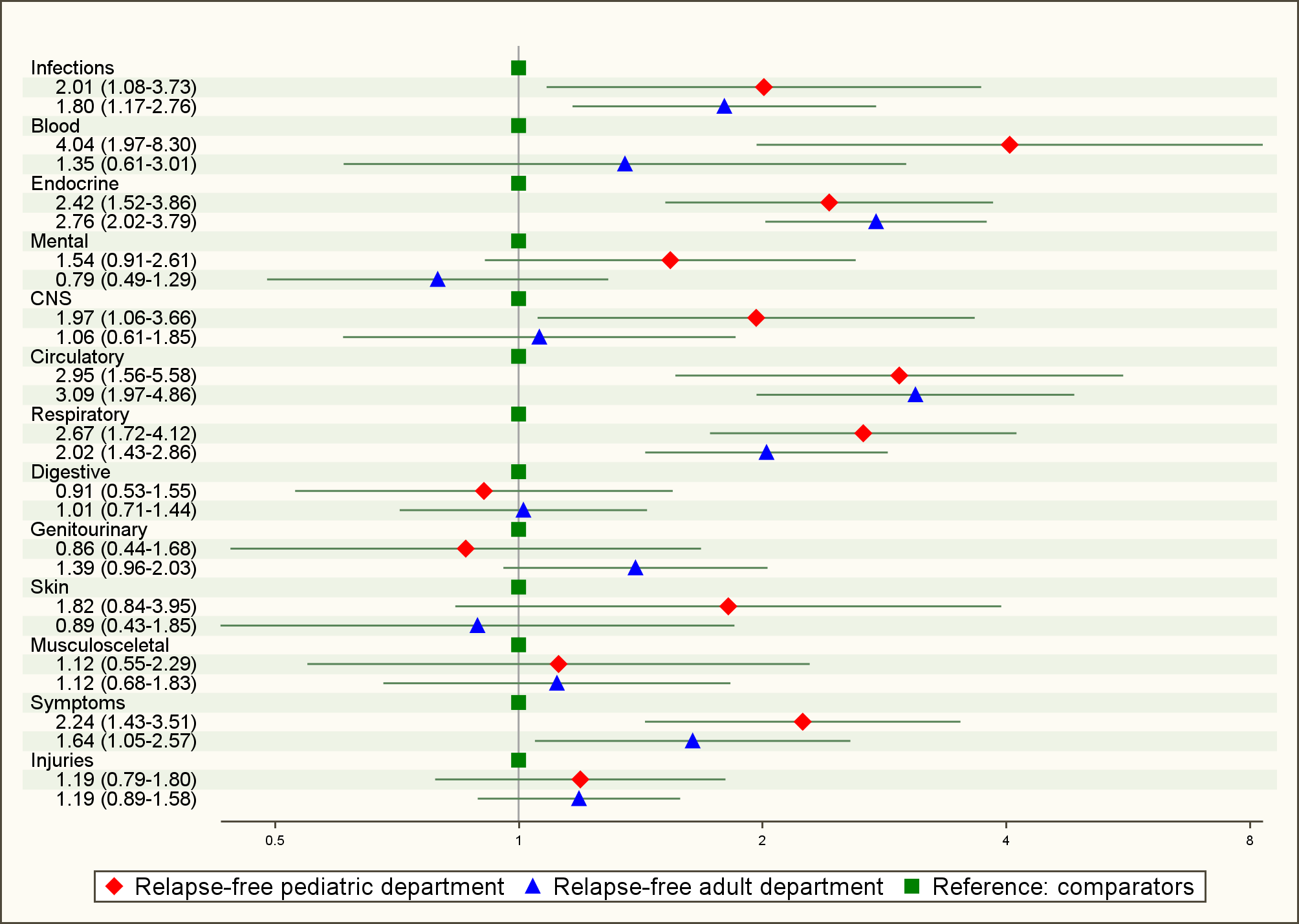

Supplement: Supplementary file 2 [file CAM4-8-4918-s002.tiff]

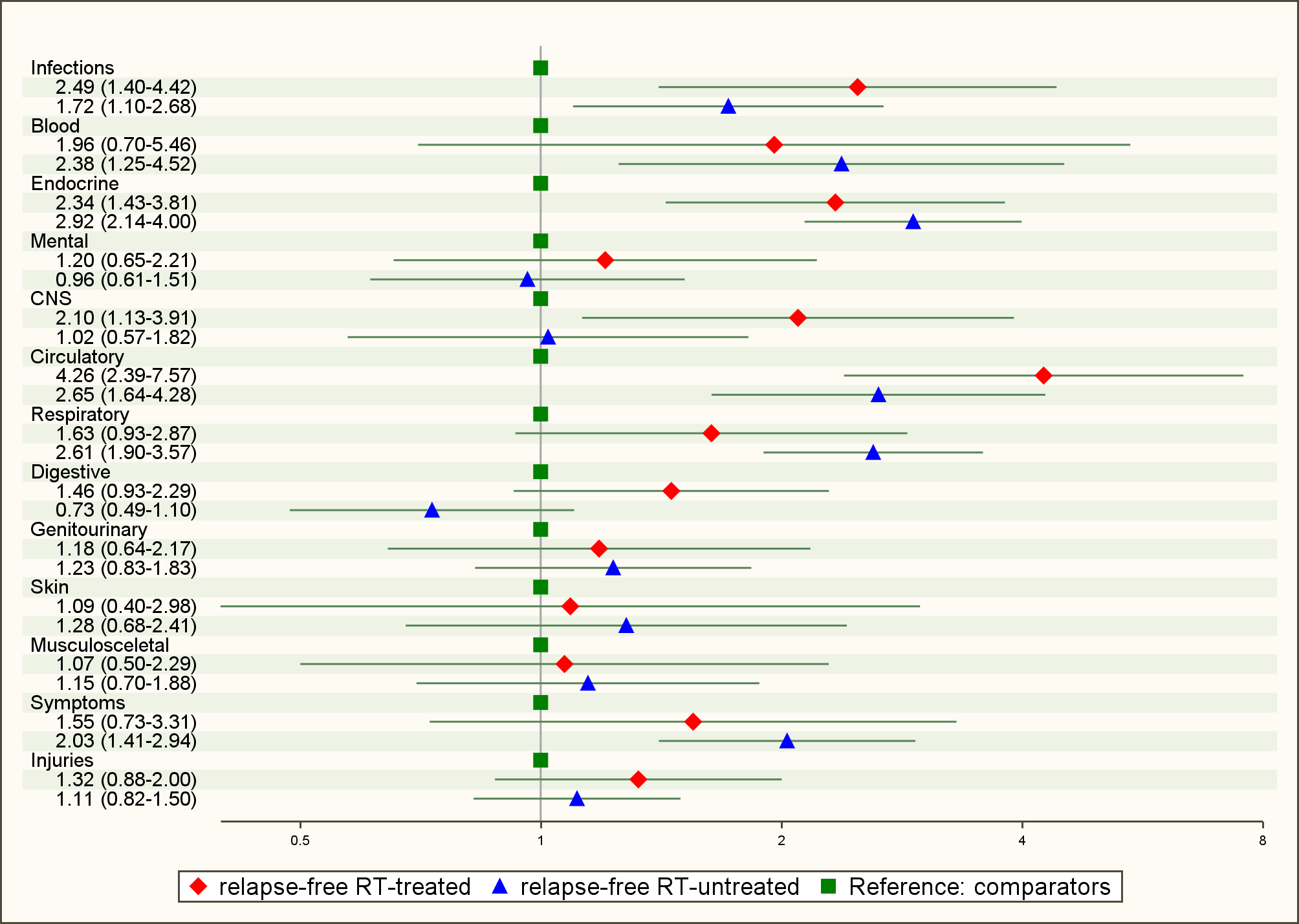

Supplement: Supplementary file 3 [file CAM4-8-4918-s003.tiff]

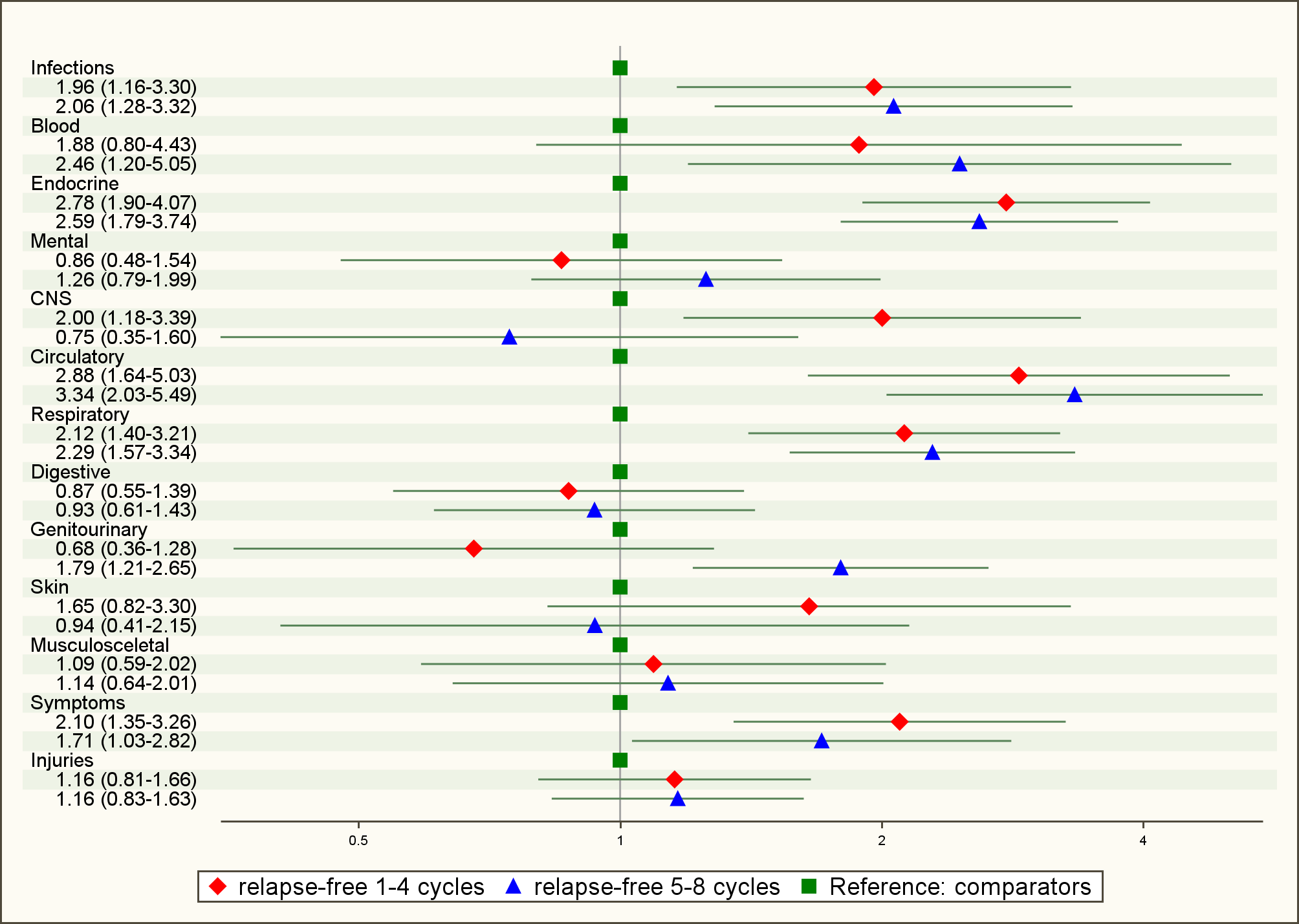

Supplement: Supplementary file 4 [file CAM4-8-4918-s004.tiff]
