# Supplementary material for: Distribution of hospital care among pediatric and young adult Hodgkin lymphoma survivors—A population‐based cohort study from Sweden and Denmark
Source: Cancer Med. 2019 Jul 2;8(10):4918–27. doi: 10.1002/cam4.2363 (PMC6712477; doi:10.1002/cam4.2363)
Supplement: Supplementary file 5 [file CAM4-8-4918-s005.docx]

**Supplemental tables**

**sTable 1. International Classification of Diseases 10th Revision (ICD-10) and associated codes^1^**

| **ICD-10 chapter** | **ICD-10 codes** | **Abbreviation** |
| --- | --- | --- |
| Neoplasms | C00 - D48*** | Neoplasms |
| Diseases of the circulatory system | I00 - I99 | Circulatory disorders |
| Infectious diseases | A00 - B99 | Infections |
| Mental and behavioural disorders | F00 - F99 | Mental |
| Diseases of the respiratory system | J00 - J99 | Respiratory |
| Diseases of the genitourinary system | N00 - N99 | Genitourinary |
| Symptoms, signs and abnormal clinical and laboratory findings, not elsewhere classified | R00 - R99 | Symptoms |
| Injury, poisoning and certain other consequences of external causes | S00 - T98 | Injuries |
| Diseases of the blood and blood-forming organs | D50 - D89 | Blood |
| Endocrine, nutritional and metabolic diseases | E00 - E90 | Endocrine |
| Diseases of the nervous system | G00 - G99 |  |
| Diseases of the eye and adnexa or diseases of the ear and mastoid process | H00 - H59 and H60 -H95 | CNS |
| Diseases of the digestive system | K00 - K93 | Digestive |
| Diseases of the skin and subcutaneous tissue | L00 - L99 | Skin |
| Diseases of the musculoskeletal system | M00 - M99 | Musculoskeletal |
| Hodgkin lymphoma* | C81.0, C81.2, C81.3, C81.7, C81.9 | Excluded from outpatient care |
| Non-Hodgkin lymphoma** | C82-C85 | Excluded from outpatient care |
| High-dose chemotherapy with stem cell transplantation**** | DR010, DR043, DR048 (autologous), DR008, DR041, DR042, DR044, DR045, DR046, DR047 (allogenic) | Relapse identification |

^1^: The chapters: pregnancy, childbirth and the puerperium (O00-O99), certain conditions originating in the perinatal period (P00-P96), congenital malformations, deformations and chromosomal abnormalities (Q00-Q99), external reasons for disease or death (V01-Y98), factors influencing health status and contact with health services (Z00-Z99) and codes for special purposes (U00-U99) were not included

* ICD-10 codes reflecting Hodgkin lymphoma were excluded from the outpatient visits analyses to eliminate registering ordinary Hodgkin lymphoma check-ups.

**ICD-10 codes reflecting non-Hodgkin lymphoma were excluded from outpatient visits analyses due to the risk of including misclassified Hodgkin lymphoma control visits.

*** Since secondary malignancies are best recorded in the Nationwide cancer registry we present these specific frequencies in the text. Visits reflecting neoplasm are although included in the overall results reflecting number of out-and inpatient visit

**** Relapse information was obtained from the registers and medical records, but to further circumvent possible underreporting, autologous and allogenic stem-cell transplantation records in the National Patient Register were also utilized.

**sTable 2.** Number underlying figure 4 and showing number of patients contributing follow-up time, total number and proportions of bed days and specialist outpatient visits for patients [relapse-free, relapsed survivors (see text for definitions)] and comparators stratified by follow-up time

| **Time since primary HL diagnosis (years)** | **0 (Dx year)** | **1-3 years** | | **4-6 years** | | **7-9 years** | | **10-12 years** | | **13+ years** | | **1+ (All years^1^)** | |
| --- | --- | --- | --- | --- | --- | --- | --- | --- | --- | --- | --- | --- | --- |
| **Number of patients** | N | N | % | N | % | N | % | N | % | N | % | N | % |
| Comparators | 4488 | 5000 |  | 4778 |  | 3815 |  | 2829 |  | 1844 |  | 5125 |  |
| Relapse-free survivors | 910 | 947 | 91% | 877 | 90% | 698 | 90% | 523 | 89% | 353 | 90% | 971 | 88% |
| Relapsed survivors | - | 93 | 9% | 98 | 10% | 80 | 10% | 64 | 11% | 40 | 10% | 123 | 12% |
|  |  |  |  |  |  |  |  |  |  |  |  |  |  |
| **Person-years of follow up (%)** |  |  |  |  |  |  |  |  |  |  |  |  |  |
| Comparators | 4370 | 13755 |  | 12860 |  | 9945 |  | 7021 |  | 8536 |  | 52117 |  |
| Relapse-free survivors | 863 | 2538 | 94% | 2354 | 91% | 1835 | 90% | 1314 | 89% | 1634 | 91% | 9676 | 91% |
| Relapsed survivors | - | 152 | 6% | 240 | 9% | 206 | 10% | 158 | 11% | 171 | 9% | 926 | 9% |
|  |  |  |  |  |  |  |  |  |  |  |  |  |  |
| **Bed days (%)** |  |  |  |  |  |  |  |  |  |  |  |  |  |
| Comparators | 528 | 3048 |  | 3791 |  | 3036 |  | 1501 |  | 1638 |  | 13022 |  |
| Relapse-free survivors | 12150 | 1451 | 41% | 959 | 45% | 870 | 77% | 678 | 78% | 742 | 77% | 4703 | 54% |
| Relapsed survivors | - | 2096 | 59% | 1181 | 55% | 255 | 23% | 189 | 22% | 218 | 23% | 3940 | 46% |
|  |  |  |  |  |  |  |  |  |  |  |  |  |  |
| **Outpatient visits (%)** |  |  |  |  |  |  |  |  |  |  |  |  |  |
| Comparators | 1455 | 5826 |  | 6491 |  | 5197 |  | 3440 |  | 4854 |  | 25819 |  |
| Relapse-free survivors | 2305 | 2453 | 86% | 2076 | 79% | 1533 | 79% | 1115 | 81% | 1328 | 89% | 8507 | 83% |
| Relapsed survivors | - | 399 | 14% | 551 | 21% | 418 | 21% | 256 | 19% | 160 | 11% | 1787 | 17% |
|  |  |  |  |  |  |  |  |  |  |  |  |  |  |
|  |  |  |  |  |  |  |  |  |  |  |  |  |  |

Abbreviations: N=Number, Dx=primary Diagnostic year. For the matched comparators diagnosis date should be interpreted as index date.

^1^ Includes all visits and bed days except during the first year of follow-up, thus the number of relapsed cases is reduced from 140 to 123
